# Therapeutic Potential of Insect Defensin DLP4 Against *Staphylococcus hyicus*-Infected Piglet Exudative Epidermitis

**DOI:** 10.3390/pharmaceutics16111350

**Published:** 2024-10-22

**Authors:** Xuanxuan Ma, Zhimin Dong, Ruoyu Mao, Xiangxue Tian, Na Yang, Weike Ren, Ya Hao, Wenluan Shen, Da Teng, Xiuli Li, Jianhua Wang

**Affiliations:** 1 Gene Engineering Laboratory, Feed Research Institute, Chinese Academy of Agricultural Sciences, Beijing 100081, China; 82101209114@caas.cn (X.M.); maoruoyu@caas.cn (R.M.); yangna@caas.cn (N.Y.); haoya@caas.cn (Y.H.); 18946678928@163.com (W.S.); 2 Innovative Team of Antimicrobial Peptides and Alternatives to Antibiotics, Feed Research Institute, Chinese Academy of Agricultural Sciences, Beijing 100081, China; 3 Key Laboratory of Feed Biotechnology, Ministry of Agriculture and Rural Affairs, Beijing 100081, China; 4 Tianjin Animal Science and Veterinary Research Institute, Tianjin 300381, Chinatiantixms@163.com (X.T.); renweike2000@126.com (W.R.); 1x15084@sina.com (X.L.)

**Keywords:** *Staphylococcus hyicus*, exudative epidermatitis (EE), insect defensin DLP4, biofilm, clinical score system

## Abstract

**Background/Objectives**: The emergence of resistance to *Staphylococcus hyicus* (*S. hyicus*), the major cause of exudative epidermatitis (EE) in piglets, has led to the need for new antimicrobial agents. The study aimed to evaluate the potential efficacy of the insect defensin DLP4 against EE in piglets caused by clinically isolated *S. hyicus* ACCC 61734. **Methods and Results**: DLP4 showed strong antibacterial activity against *S. hyicus* ACCC 61734 (minimum inhibitory concentration, MIC: 0.92 μM, median effect concentration, EC_50_: 3.158 μM). DLP4 could effectively inhibit the formation of *S. hyicus* early biofilm with an inhibition rate of 95.10–98.34% and eradicate mature biofilm with a clearance rate of 82.09–86.41%, which was significantly superior to that of ceftriaxone sodium (CRO). Meanwhile, DLP4 could efficiently inhibit bacteria in early and mature biofilm, killing up to 95.3% of bacteria in early biofilm and 87.2–90.3% of bacteria in mature biofilm. The results showed that DLP4 could be effective in alleviating the clinical symptoms of EE by down-regulating the nuclear factor κB (NF-κB) signaling pathway, balancing cytokines, inhibiting bacterial proliferation, and reducing organ tissue damage. **Conclusions**: This study firstly demonstrated the potential efficacy of DLP4 against EE caused by *S. hyicus* ACCC 61734 infection in piglets, which may be used as an alternative to antibiotics in treating EE.

## 1. Introduction

Exudative epidermatitis (EE), also known as “greasy pig disease” [1,2], commonly in 1–5 weeks of piglets, for its typical clinical symptoms: in the early days, occurrence of erythema of the ear, face, abdomen, skin leaking slurry and fall off form the outermost layer of skin, then spread to the whole body, showing rough coat and black back [3]. EE can be divided into acute and sub-acute according to the characteristics of the disease process. The onset of acute EE occurs 3–6 days after infection and causes the death of piglets. Subacute usually has a low mortality rate, and sick pigs grow slowly after recovery and form “stiff pigs” [1]. *Staphylococcus hyicus* (*S. hyicus*) is widely recognized as the major cause of EE in piglets [4,5]. Its secretion of epidermal exfoliative toxins (ExhA, ExhB, ExhC, ExhD, ShetA, and ShetB) acts specifically on desmoglein 1 (Dsg1) to cause separation of keratinocytes, resulting in blistering of the skin to form dermatitis, which is in line with the mechanism of staphylococcal scalded skin syndrome (SSSS) caused by the exfoliative toxins (ETA, ETB and ETD) of *S. aureus* [6,7,8]. Recently, the worldwide outbreak of EE has brought huge economic losses to animal husbandry, and the prevention and control of EE have increasingly received widespread attention [9,10].

Antibiotics are the mainstay of treatment for EE in piglets, but the irrational use of antibiotics has resulted in increasing antibiotic resistance and the emergence of multi-drug resistant (MDR) bacteria [11,12]. According to reports, the resistance rates of *S. hyicus* isolated from 30 pig herds in Canada to penicillin G, ampicillin, ceftiofur, tetracycline, spectinomycin, and tilmicosin were 97.2%, 97.2%, 71.1%, 55.6%, 45.1% and 31.0%, respectively [13]. The resistance rates to penicillin, tetracycline, erythromycin, and ampicillin among 196 strains of *S. hyicus* isolated from pig farms in Fujian (China) were 83.2%, 75.5%, 74.5%, and 74.0%, respectively [14]. In this study, clinically isolated *S. hyicus* ACCC 61734 was resistant to penicillin, sulfamethoxazole, chloramphenicol, streptomycin, azithromycin, erythromycin, tetracycline, mycotoxins and norfloxacin, and treatment of *S. hyicus* infections is challenging [2]. Meanwhile, the biofilm formed by *Staphylococcus* spp. confers bacterial resistance to both immune defenses and antibiotics [15,16], which may further exacerbate the antibiotic resistance crisis and the difficulty in preventing and controlling EE. Therefore, there is an urgent need for novel and effective antimicrobials that can eradicate MDR biofilm and promote healing in EE.

As an important component of innate immunity, antimicrobial peptides (AMPs) widely exist in microorganisms, animals, and plants, and their highly potent antimicrobial activity and unique antimicrobial mechanism have attracted much attention and are considered to be one of the reserve antimicrobial drugs with a wide range of application prospects [17]. The current research on AMPs has reached a consensus that AMPs are more suitable for local treatment than systemic drugs, which has been verified in the clinical transformation of AMPs, and most of the AMPs in clinical research are applied to diabetic hand-foot-mouth disease and skin wound infection [18], and a large number of studies have shown that the AMPs (LL-37, cecropin A, NZ2114, and N6) can effectively eradicate biofilm [19,20,21,22], which indicates a great application prospect of AMPs in biofilm inhibition and wound healing of skin infection [23,24]. The AMP DLP4 in this study was derived from the *Hermetia illucens*, and its MIC against gram-positive bacteria (*Staphylococcus aureus*, *Staphylococcus epidermidis*, *S. hyicus,* and *Staphylococcus suis*) was less than 1.88 μM, the hemolysis rate (128 μg/mL) was less than 1.46% and was not easy to develop resistance [25], and in vivo, it could effectively alleviate mouse skin abscesses by inhibiting bacterial proliferation and regulating cytokines [2,26]. Based on our previous study on the antibacterial activity and mechanism of DLP4 against *S. hyicus* [25], we investigated the anti-biofilm activity of DLP4 against *S. hyicus* ACCC 61734 in vitro, established the piglet EE model caused by *S. hyicus* infection, and systematically evaluated the potential therapeutic effect of DLP4 on EE in piglets.

## 2. Materials and Methods

### 2.1. Materials and Strains

*S. hyicus* NCTC 10350 was purchased from the National Collection of Type Culture (NCTC, London, UK). Clinical strain *S. hyicus* ACCC 61734 was isolated from the kidney of a piglet with defined EE by Tianjin Animal Science and Veterinary Research Institute. *S. epidermidis* ATCC 35984 and *S. epidemidis* ATCC 12228 were purchased from the American Type Culture Collection (ATCC, Manassas, VA, USA). *Staphylococcus sciuri* FRI 18 and *S. sciuri* FRI 5 were isolated from cows with mastitis in Tianjin in 2017 by the Gene Engineering Laboratory of Feed Research Institute. Insect defensin DLP4 (with purity over 90%) was prepared as described in our previous study [25]. Eight-day-old healthy piglets with no history of EE were purchased from Tianjin Nongkang Breeding Co., Ltd. (Wuqing District, Tianjin, China). All other chemical reagents used were of analytical grade. 

### 2.2. Antimicrobial Activity Determination

The Minimum inhibitory concentrations (MICs) of insect defensin DLP4 against *S. hyicus* were determined according to the Clinical and Laboratory Standards Institute (CLSI) microdilution method [27]. The specific methods were as follows: (1) Strain preparation: the test strain was activated, 1% was transferred and incubated to the logarithmic growth phase, and diluted to 1 × 10^5^ CFU/mL with culture medium for later use; (2) antibacterial drug dilution: each antibacterial drug was dissolved in PBS, and then diluted by two-fold dilution method; (3) 90 µL of bacterial suspension and 10 µL of serial two-fold dilutions of peptide (5–1280 µg/mL) were added to 96-well plates and incubated for 18 h at 37 °C. Three replicates were used for each treatment.

### 2.3. Dose-Killing Curve

The *S*. *hyicus* cells were diluted to 1 × 10^5^ CFU/mL, and then different concentrations of DLP4 were added to 96-well plates with final concentrations of 3/64, 1/16, 3/32, 1/4, 3/8, 1/2, 1/2, 3/4, 1, 2, 4, 6, 8, 12, 16 × MIC. Then incubated for 24 h at 37 °C, the samples were taken for plate counting and to construct a dose-response curve [28]. The PBS group served as the negative control.

### 2.4. Biofilm Formation Assay

The biofilm formation ability of *S. hyicus* ACCC 61734 and *S. hyicus* NCTC 10350 was analyzed by crystal violet staining [21]. *S. epidermidis* ATCC 35984 and *S. epidermidis* ATCC 12228 were used as positive and negative strains for biofilm formation ability assay, respectively. The specific procedures were as follows: (1) The test strain (1 × 10^8^ CFU/mL, 200 µL) was inoculated in 96-well plates and incubated for 24 h, and the TSB medium was used as blank control. (2) After incubation, the biofilm was rinsed with PBS and dried naturally, then fixed with 2.5% glutaraldehyde for 1.5 h and stained with 0.1% crystal violet for 15 min. (3) After staining, the biofilm was immersed in 95% ethanol for 30 min and determined the absorbance at 570 nm using a microplate reader. All experiments were replicated three times. The biofilm formation ability of the strain was determined according to the absorbance value at 570 nm.

### 2.5. Effect of DLP4 on Early Biofilm of S. hyicus

To assess the inhibitory effect of DLP4 on early biofilm formation of *S. hyicus*, test strains (1 × 10^8^ CFU/mL, 180 µL) were inoculated in 96-well plates, DLP4 (2–128 µg/mL, 20 µL) was added to the 96-well plates and then incubated for 24 h. Biofilm formation was assessed by crystal violet staining. Untreated bacteria were used as negative control (A), and fresh TSB broth acted as blank control (A_0_). The inhibitory activity of DLP4 against early biofilm was calculated using the following equation: Biofilm mass (%) = [(A_peptide_ − A_0_)/(A − A_0_)] × 100% [21]. All experiments were conducted in triplicate.

### 2.6. Effect of DLP4 on Mature Biofilm of S. hyicus

To assess the effect of DLP4 on mature *S. hyicus* biofilm, test strains (Log-phase, 1 × 10^8^ CFU/mL) were seeded into 96-well plates and incubated for 24 h, and different concentrations of DLP4 (2–256 µg/mL) were added and incubated for 24 h. The efficacy of DLP4 in eliminating mature biofilm was determined using the crystal violet staining method [21]. The calculation formula for mature biofilm mass (%) was the same as Section 2.5. Ceftriaxone sodium (CRO) was used as the antibiotic control. All experiments were conducted in triplicate.

### 2.7. Effect of DLP4 on Bacteria in Early Biofilm

The test strains (Log-phase 1 × 10^8^ CFU/mL) were inoculated into a 96-well plate and incubated for 24 h. After washing with PBS, different concentrations of DLP4 (2–256 µg/mL) were added and incubated for 2 h. PBS was used as the blank control. After incubation, the bacterial solution was sonicated for 5 min to disrupt the biofilm and facilitate cell release, followed by colony plate counting [21]. CRO was used as the antibiotic control. All experiments were replicated three times.

### 2.8. Effect of DLP4 on Persister in Mature Biofilm

To study the effect of DLP4 on *S. hyicus* persister in mature biofilm, test strains (Log-phase, 1 × 10^8^ CFU/mL) were seeded into 96-well plates and incubated for 24 h. Following incubation, the plates were washed three times with 0.01 mol/L PBS to remove planktonic bacteria. Then, vancomycin (100 × MIC, 200 µL) was added and incubated for 24 h. After washing with PBS, DLP4 (32 × MIC, 200 µL) was added and incubated for 24 h. The bacterial suspension was sonicated for 5 min, followed by colony plate counting [29]. CRO was used as the antibiotic control. All experiments were performed in triplicate.

### 2.9. Effect of DLP4 on EE Induced by S. hyicus in Piglets

#### 2.9.1. Piglets Exudative Dermatitis Treatment

To study the therapeutic effects of DLP4 on target animals in vivo, a total of 30 cross-breed piglets (Danish Landrace, Danish Yorkshire, and Duroc, 10-day-old) with no history of EE infection were randomly divided into 6 groups: blank control group (CK: uninfected and untreated), negative control group (NC: infected but untreated), DLP4 treatment groups (40 mg/kg and 20 mg/kg body weight, 1 mL), and CRO treatment groups (40 mg/kg and 20 mg/kg body weight, 1 mL). The experiment lasted for 10 days. *S. hyicus* ACCC 61734 (1 × 10^9^ CFU/mL, 1 mL) was injected subcutaneously into the posterior neck of the right ear [30]. After 24 h of infection, the corresponding drugs were injected intramuscularly according to the above groups for 5 consecutive days. During the test, clinical signs were photographed daily. The animal study was approved by the Animal Care and Use Committee of the Feed Research Institute of the Chinese Academy of Agricultural Sciences (FRI-CAAS-20200919).

#### 2.9.2. Sample Collection

Blood samples were collected via the anterior vena cava on days 1, 4, and 9 after infection, and serum and whole blood were collected. Piglets were executed harmlessly at 9 d post-infection and dissected under aseptic conditions to isolate the ear skin, spleens, kidneys, lungs, and livers for subsequent testing.

#### 2.9.3. Establishment of Scoring System for EE in Piglets

**Figure 1:**
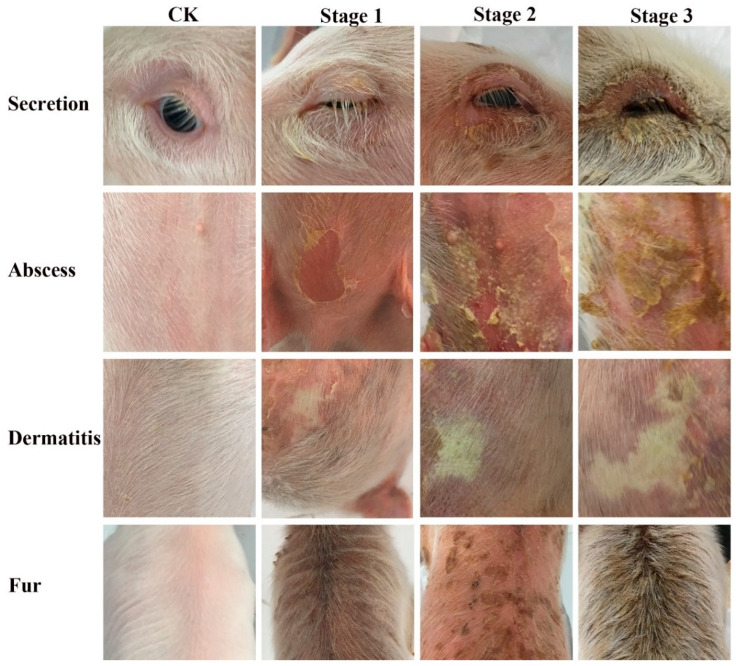
Scoring criteria for the intensity of EE in piglets. The secretion, dermatitis symptoms, abscess, and fur status were scored. Stages 1, 2, and 3 represent 1, 2, and 3 points, respectively. The stage 2 photo of the abscess and the stage 3 photo of Fur were from the results inclduing partial photo sources in Section 3.8.1.

All groups of piglets were monitored and photographed throughout the trial for further scoring. Due to the lack of criteria for evaluating the severity of EE, to ensure a fair and credible evaluation of efficacy, we established scoring criteria for EE in piglets based on the SCORAD scoring system [31], including the spread area score A (Table 1) and the dermatitis severity score B (Figure 1). A = lesion spread (100); B = intensity (12). Total score = A/5 + 7B/2 (The maximum grand is 62; The comprehensive evaluation coefficient was referred to as SCORAD. Clinical signs were then independently scored by three researchers according to the clinical scoring system.

#### 2.9.4. Organ Colony Counts

To assess the in vivo therapeutic efficacy of DLP4 in EE, the kidneys, lungs, livers, and spleens of piglets were collected and weighed; after tissue homogenization, the organ bacterial load was determined by plate counting method, and the blood colony was also counted [2].

#### 2.9.5. Inflammatory Factor Measurement

The serum levels of tumor necrosis factor (TNF-α), interleukin IL-6, IL-10, IL-12p40p70, and granulocyte-macrophage colony-stimulating factor (GM-CSF) were determined by using ELISA kits (Nanjing Jian cheng Bioengineering Institute, Nanjing, China) [5]. In brief, the blood was collected from the anterior vena cava in a procoagulant tube, centrifuged at 3000 rpm for 10 min at 4 °C, and the serum was collected for ELISA analysis.

#### 2.9.6. Western Blot Analysis

Total proteins were extracted from skin tissue homogenates, and the protein concentration of the supernatant was quantified using the Bradford Protein Assay Kit; 10% sodium dodecyl sulfate-polyacrylamide gel electrophoresis was used to separate proteins and transferred to nitrocellulose membranes. The supernatants were then incubated with primary antibodies (TLR2, IκB, p-IκB, p65, and p-p65) overnight at 4 °C; after washing, secondary antibodies were added and incubated for 30 min. Relative protein expression levels were quantified by optical density measurements of chemiluminescent reaction bands. β-actin was used as an internal control [26].

#### 2.9.7. Histopathological Analysis

The collected skin, lung, and kidney tissues were rinsed in pre-cooled PBS and placed in 4% paraformaldehyde for fixation overnight. Complete sections were then obtained after dehydration, wax immersion, sectioning, dewaxing, hematoxylin-eosin (HE) staining, and sealing. The piglets were examined under a light microscope to assess the extent of skin and organ damage [5].

### 2.10. Statistical Analysis

All data were statistically analyzed using GraphPad Prism software v9.0 (GraphPad Software, La Jolla, CA, USA) and expressed as mean ± standard deviation (SD). The statistical significance of the difference between the groups was determined using the single-factor analysis of variance and Tukey multiple comparisons analysis.

## 3. Results

### 3.1. MIC Determination

Biological activity is a prerequisite for evaluating the application of AMPs, and the antimicrobial activity of DLP4 was first evaluated by MIC. The antibacterial activity of DLP4 against *S. hyicus* NCTC 10350 and clinical isolates *S. hyicus* ACCC 61734, *S. sciuri* FRI 18, and *S. sciuri* FRI 5 was determined by the MIC assay. As shown in Table 2, the MIC of DLP4 against *S. hyicus* NCTC 10350 and *S. hyicus* ACCC 61734 was 0.92 μM, and the MIC of DLP4 against *S. sciuri* FRI 18 and *S. sciuri* FRI 5 was 0.46 μM, which were significantly lower than those of CRO (1.51–12.08 μM) and ceftiofur (1.51–12.08 μM). The results indicate that DLP4 had more potent antibacterial activity towards *S. hyicus* and *S. sciuri*, which is consistent with the results of previous studies [2,5,25].

### 3.2. Dose Killing Curve

**Figure 2:**
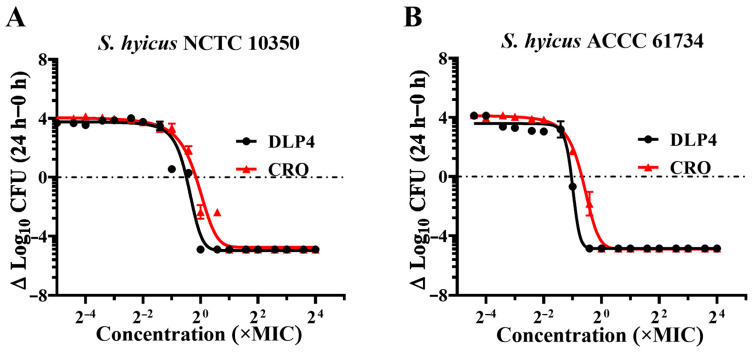
In vitro antibacterial activity. (**A**) Dose killing curves of DLP4 and CRO against *S. hyicus* NCTC 10350 in vitro. (**B**) Dose killing curves of DLP4 and CRO against *S. hyicus* ACCC 61734 in vitro. Results were given as mean ± SD (*n* = 3).

Besides MIC, bactericidal kinetics is an important indicator of AMPs’ biological activity. The strains *S. hyicus* NCTC 10350 and *S. hyicus* ACCC 61734 were incubated with different concentrations of DLP4 or CRO for 24 h to count colonies and draw a dose-dependent bactericidal curve. As shown in Figure 2 and Table 3, both DLP4 and CRO displayed obvious dose-dependent effects on *S. hyicus* NCTC 10350 and *S. hyicus* ACCC 61734. The curve and its corresponding parameters of DLP4 against *S. hyicus* NCTC 10350 showed a leftward shift compared to those of CRO, with Emax values of −4.966 and −4.752 Log_10_ CFU/mL, and the EC_50_ of the DLP4 and CRO groups were 5.580 and 8.413 μM, respectively. The curve and its corresponding parameters of DLP4 against *S. hyicus* ACCC 61734 showed that compared with CRO, the curve of the DLP4 group was shifted to the left, the Emax values were −4.855 and −4.891 Log_10_ CFU/mL, and the corresponding EC_50_ were 3.158 and 4.406 μM, respectively. The above results show that DLP4 exhibits superior bactericidal potential against *S. hyicus* NCTC 10350 and *S. hyicus* ACCC 61734 than those of CRO. Meanwhile, the hill coefficient (the slope of the pharmacodynamic curve) describes the window between no effect and the concentration that leads to complete killing. The results showed that the hill coefficients of DLP4 against *S. hyicus* were superior to that of CRO, indicating reduced selective pressure for bacterial resistance to DLP4 [32].

### 3.3. Biofilm Formation Ability

**Figure 3:**
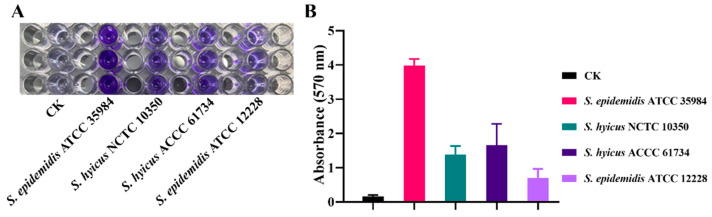
Crystal violet staining method of biofilm identification. (**A**) Identification of *S. hyicus* biofilm forming ability by crystal violet staining. CK: PBS group (**B**) Quantification of biofilm formation capacity of *S. hyicus* by crystalline violet staining. Results were given as mean ± SD (*n* = 3).

Bacteria accumulate in the extracellular matrix and form biofilm, which acts as a barrier against antimicrobial agents. Therefore, it is necessary to evaluate the ability of bacterial biofilm formation firstly by crystal violet staining. According to the evaluation criteria of biofilm-forming ability by crystal violet staining, the OD_570_ nm absorbance values of *S. hyicus* NCTC 10350 and *S. hyicus* ACCC 61734 were 1.38 ± 0.23 and 1.65 ± 0.60, respectively, which were nearly 8.5–10.4-fold higher than that of the CK group (0.16 ± 0.04) (Figure 3). According to the biofilm formation criteria, both *S. hyicus* NCTC 10350 and *S. hyicus* ACCC 61734 were strong film-forming strains.

### 3.4. Inhibition Effect of DLP4 on Early Biofilm of S. hyicus

**Figure 4:**
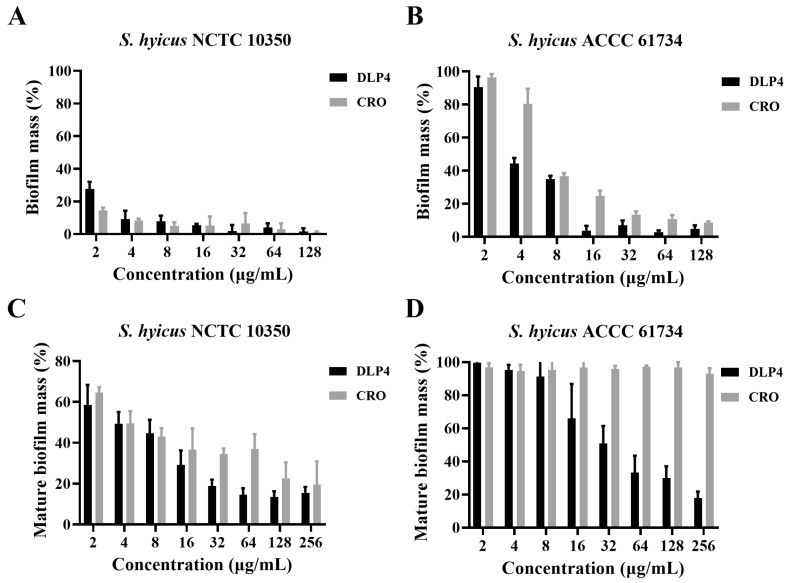
Effects of DLP4 on *S. hyicus* biofilm. (**A**) Inhibition effect of DLP4 on biofilm formation of *S. hyicus* NCTC 10350. (**B**) Inhibition effect of DLP4 on biofilm formation of *S. hyicus* ACCC 61734. (**C**) Eradication effect of DLP4 on *S. hyicus* NCTC 10350 mature biofilm. (**D**) Eradication effect of DLP4 on *S. hyicus* ACCC 61734 mature biofilm. Results were given as mean ± SD (*n* = 3).

DLP4 and antibiotics were added at the early stage of biofilm formation to study the inhibition effect of DLP4 on the early biofilm of *S. hyicus*. The results are shown in Figure 4A,B. DLP4 inhibited the biofilm formation of *S. hyicus* NCTC 10350 and *S. hyicus* ACCC 61734 in a concentration-dependent manner. After treatment with 2–128 µg/mL DLP4 for 24 h, the inhibition rate of DLP4 against *S. hyicus* NCTC 10350 early biofilm was 72.34–98.34% (Figure 4A), and the inhibition rate of DLP4 against *S. hyicus* ACCC 61734 early biofilm ranged from 9.55% to 95.10% (Figure 4B), which were comparable to that of CRO group (3.45–99.07%).

### 3.5. Eradication Effect of DLP4 on Mature Biofilm of S. hyicus

To further evaluate the ability of DLP4 to eliminate bacterial biofilm, DLP4, and antibiotics were added after mature biofilm formation to investigate the inhibitory effect of DLP4 on the mature biofilm of *S. hyicus*. The results, as shown in Figure 4C,D, and DLP4, were able to remove the mature biofilm of *S. hyicus* NCTC 10350 and *S. hyicus* ACCC 61734 in a dose-dependent manner. After treatment with 2–256 µg/mL DLP4 for 24 h, the eradication rate of the mature biofilm of *S. hyicus* NCTC 10350 was 41.34–86.41% (Figure 4C), and the inhibition rate of mature biofilm of *S. hyicus* ACCC 61734 was 0.33–82.09% (Figure 4D). However, the effect of CRO on mature biofilm was poor, especially for *S. hyicus* ACCC 61734 (256 µg/mL: 7.03%). These results indicate that DLP4 has the potential to eradicate mature biofilm within a range of concentrations.

### 3.6. Bactericidal Effect of DLP4 on Bacteria in Early Biofilm

**Figure 5:**
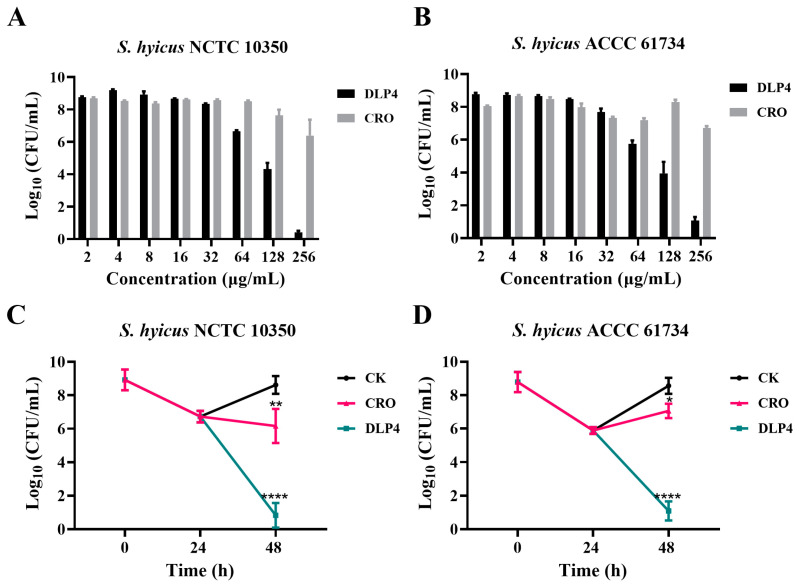
Bactericidal activity of DLP4 on *S. hyicus* biofilm bacteria. (**A**) Bactericidal activity of DLP4 against established biofilm of *S. hyicus* NCTC 10350. (**B**) Bactericidal activity of DLP4 against established biofilm of *S. hyicus* ACCC 61734. (**C**) Bactericidal activity of DLP4 against persisters from *S. hyicus* NCTC 10350 mature biofilm. (**D**) Bactericidal activity of DLP4 against persisters from *S. hyicus* ACCC 61734 mature biofilm. Results were given as mean ± SD (*n* = 3). *: *p* < 0.05; **: *p* < 0.01; ****: *p* < 0.0001.

The bactericidal activity of DLP4 against biofilm bacteria was determined by plate counting. As shown in Figure 5A,B, DLP4 reduced the load of *S. hyicus* in biofilm in a dose-dependent manner. The bactericidal rate of DLP4 (32–256 µg/mL) towards *S. hyicus* NCTC 10350 in early biofilm was 5–95.3%, and the corresponding rate against *S. hyicus* ACCC 61734 was 12.4–88.0%, whereas 2–16 µg/mL DLP4 had less effect on bacteria in early biofilm. Meanwhile, the bactericidal rate of CRO (256 µg/mL) treatment on *S. hyicus* ACCC 61734 in early biofilm was only 22.3%, which was significantly less than that of DLP4, indicating that DLP4 was more effective than CRO in killing bacteria in the biofilm.

### 3.7. Bactericidal Effect of DLP4 on Persister in Mature Biofilm

The bacteria were artificially induced to produce persisters through the external application of antibiotics to further evaluate the inhibitory effect of DLP4 on persisters in mature biofilm. As shown in Figure 5C,D, after 100 × MIC vancomycin treatment for 24 h, there were still 7.68 × 10^5^–5.33 × 10^6^ CFU/mL of persistent bacteria in mature biofilm. The bactericidal rates of DLP4 (32 × MIC) against *S. hyicus* NCTC 10350 and *S. hyicus* ACCC 61734 in mature biofilm reached 90.3% and 87.2%, respectively, which were significantly higher than those of the CRO group (29.1% and 17.7%).

### 3.8. Piglet In Vivo Experiments

#### 3.8.1. Observation of Clinical Symptoms of EE

**Figure 6:**
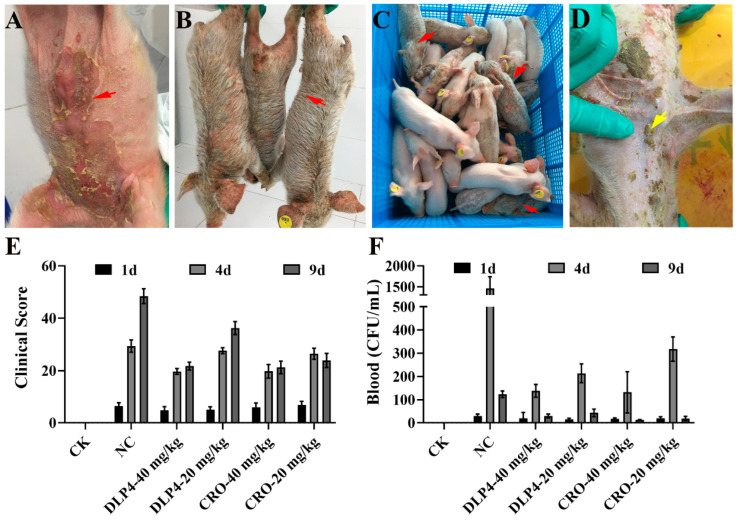
The therapeutic effect of DLP4 in piglet EE induced by *S. hyicus* ACCC 61734. (**A**–**D**) Clinical observation of piglets with EE. (**A**) Typical clinical symptoms of piglets in the untreated group after 3 days (d) of infection with *S. hyicus* ACCC 61734. (**B**) Typical clinical symptoms of piglets in the untreated group after 9 days of infection with *S. hyicus* ACCC 61734. (**C**) Comparison of clinical manifestations in each treatment group. (**D**) The clinical symptoms of infected piglets were relieved after 5 days of continuous treatment with DLP4. Red arrow: symptoms of untreated piglets: skin exfoliation, brown fluid exudation, rough coat; yellow arrow: skin healing symptoms of piglets in the treatment group. (**E**) Piglet clinical symptom score after infection for 1 d, 4 d, and 9 d. (**F**) Effect of DLP4 and CRO on blood bacterial burdens in piglets after infection for 1 d, 4 d, and 9 d. Red arrow: typical symptoms of EE, yellow arrows: recovery symptoms of EE.

After infection with *S. hyicus* ACCC 61734 for 1 d, all piglets started to show clinical symptoms of EE, starting to present with redness and EE only in the inoculation site and then rapidly spreading to the abdominal and facial skin and even the whole body. Infected piglets were characterized by blister formation, skin exfoliation, and brown fluid exudation (Figure 6). As shown in Figure 6A, infected and untreated piglets showed large pieces of epidermis peeled away, leaving extensive areas of sensitive and painful bare skin, which further caused fluid loss, worsening infection, renal punctate hemorrhage, and pulmonary fibrosis. However, there was significant relief in symptoms after treatment with DLP4 or CRO (Figure 6D), which manifested specifically as the skin began to scab (Figure 6D). To evaluate dermatitis symptoms effectively further, piglets were scored according to lesion extent and intensity; DLP4 and CRO treatment all delayed the progression of the disease. As shown in Table 4 and Figure 6, the clinical symptom scores of EE in piglets in the NC group, PC group, CRO (40 mg/kg), CRO (20 mg/kg), DLP4 (40 mg/kg), and DLP4 (20 mg/kg) were 0, 48.467, 21.716, 36.233, 21.231 and 23.897, respectively. The results showed that *S. hyicus* ACCC 61734 infection successfully caused EE in piglets, and both CRO and DLP4 treatment groups were effective in alleviating the clinical symptoms of dermatitis in piglets, and DLP4 (40 mg/kg) achieved the best therapeutic effect. Meanwhile, the scores of different individuals did not show much difference, indicating the effectiveness of the scoring system.

#### 3.8.2. Inhibition of Bacterial Translocation

**Figure 7:**
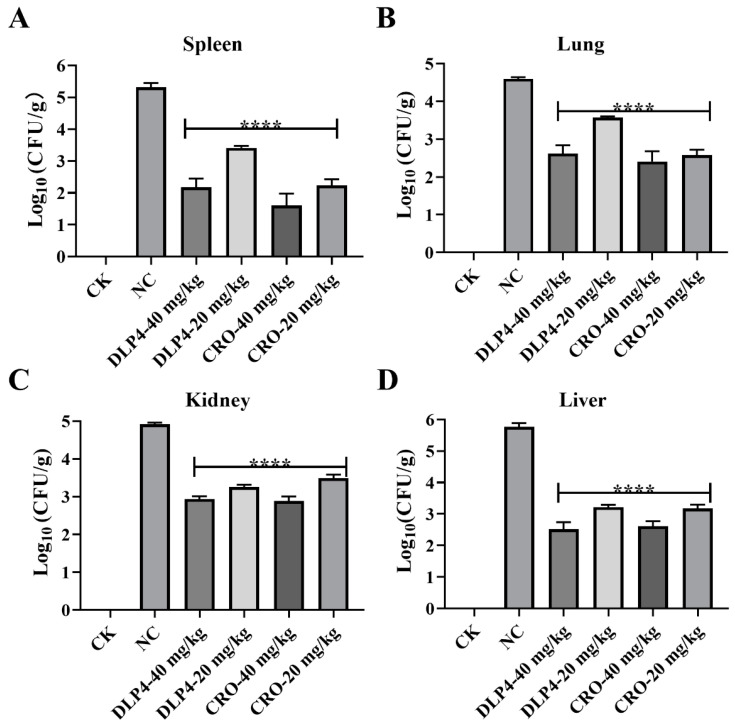
Protective effects of DLP4 on piglets with EE. (**A**) Effect of DLP4 and CRO on lung bacterial burdens in piglet after infection for 9 d. (**B**) Effect of DLP4 and CRO on spleen bacterial burden in piglet after infection for 9 d. (**C**) Effect of DLP4 and CRO on kidney bacterial burdens in piglet after infection for 9 d. (**D**) Effect of DLP4 and CRO on liver bacterial burdens in piglet after infection for 9 d. ****: *p* < 0.0001.

To study the proliferation and distribution of *S. hyicus* ACCC 61734 in vivo, the blood bacterial load of *S. hyicus* ACCC 61734 was determined on days 1, 4, and 9. As shown in Figure 6F. On day 1, the blood bacterial load was less than 10^2^ CFU/mL, and on day 4, the blood load of the NC group was 1.46 × 10^3^ CFU/mL, which was significantly higher than those of the other treatment groups (1.32–3.18 × 10^2^ CFU/mL), and on day 9, loads of all treatment groups were less than 10^2^ CFU/mL, which was significantly less than that of the NC group (1.23 × 10^2^ CFU/mL), suggesting that *S. hyicus* ACCC 61734 caused systemic infection in piglets by entering the bloodstream. DLP4 and CRO treatment can effectively reduce blood bacterial load. To further verify the distribution of *S. hyicus* ACCC 61734 in the organs after systemic infection, the spleens, lungs, kidneys, and livers of the piglets were collected for colony counting. As shown in Figure 7, on day 9, the bacteria load of the spleen, lung, kidney, and liver in the NC group was 6.31 × 10^5^, 1.97 × 10^5^, 3.30 × 10^5,^ and 2.93 × 10^6^ CFU/g, respectively. After treatment with DLP4, the corresponding bacteria load was significantly reduced by 1.12–3.35 Log_10_ CFU/g, which was comparable to the effect of the CRO group (1.43–3.15 Log_10_ CFU/g).

#### 3.8.3. Modulation of Inflammatory Cytokines

**Figure 8:**
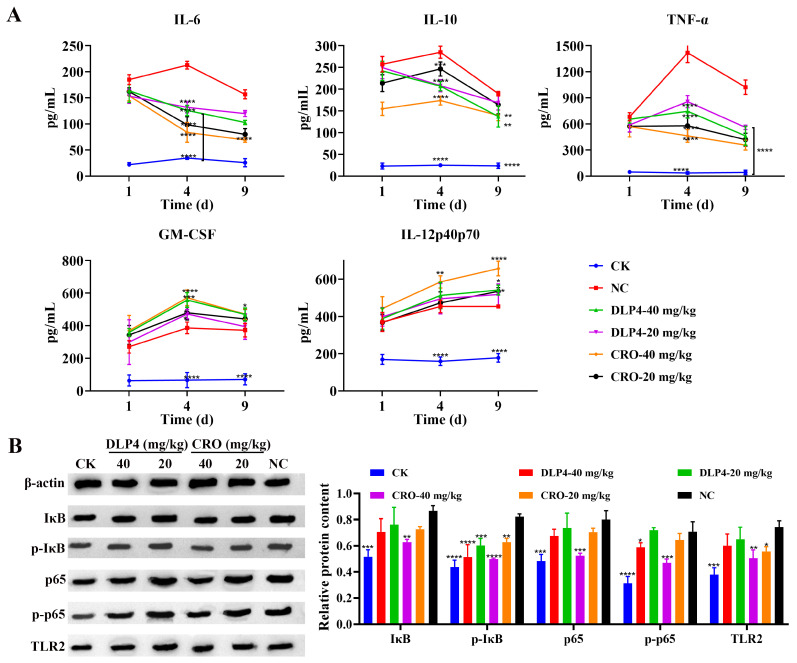
The efficacy of DLP4 on serum cytokine and TLR2-NF-κB signaling pathway. (**A**) Piglets were subcutaneously injected with *S. hyicus* ACCC 61734 and followed by treatment with DLP4 and CRO for 5 consecutive days. Serum was collected to detect cytokines (IL-6, IL-10, TNF-α, GM-CSF, and IL-12p40p70) at 1 d, 4 d, and 9 d after infection, respectively. (**B**) Effects of DLP4 on TLR2-NF-κB signaling (*n* = 3). The statistical significance of differences between experimental and negative control (PBS) was determined using one-way ANOVA and Dunnett’s multiple comparisons. * *p* < 0.05; ** *p* < 0.01; *** *p* < 0.001; ****: *p* < 0.0001.

To further investigate whether the protective effects of DLP4 on piglets were related to the regulation of inflammatory factors, the serum levels of IL-6, IL-10, TNF-α, GM-CSF, and IL-12p40p70 were measured. As shown in Figure 8A, compared with the levels of inflammatory factors in the untreated group, the levels of IL-6, IL-10, and TNF-α in the DLP4 (40 mg/kg) treatment group decreased at 34.5%, 27.6%, and 54.7%, respectively, which were comparable to those of CRO (40 mg/kg) treatment group (55.8%, 26.3%, and 65.2%, respectively). However, the levels of IL-12p40p70 and GM-CSF in the DLP4 (40 mg/kg) treatment group were increased by 25.9% and 19.4%, respectively, compared to the corresponding control group, and the CRO (40 mg/kg) treatment group were increased by 26.4% and 44.9%, respectively. These results revealed that DLP4 could reduce the production of IL-6, IL-10, and TNF-α and promote the synthesis of IL-12p40p70 and GM-CSF.

#### 3.8.4. NF-κB Signaling Pathway Analysis

TLR2 is a key component of the host innate immune system, which can recognize microbial-related pathogenic molecules, mediate the production of inflammatory cytokines, and lead to the activation of the NF-κB signaling pathway. To study the anti-inflammatory mechanism of DLP4, the expression of IκB, p-IκB, p65, p-p65, and TLR2 was analyzed by western blot. As shown in Figure 8B, the expression of TLR2, IκB, p-IκB, p65, and p-p65 was significantly increased in the NC group compared with that in the CK group. After treatment with DLP4, the expression levels of TLR2, IκB, p-IκB, p65, and p-p65 were decreased to varying degrees, in which the expression of p-IκB and p-p65 was significantly reduced in DLP4 (40 mg/kg) group, the effect was slightly inferior to that of CRO group. The above results indicated that DLP4 could inhibit the expression of p-IκB and p-p65 and down-regulate the NF-κB pathway.

#### 3.8.5. Histopathology Analysis

**Figure 9:**
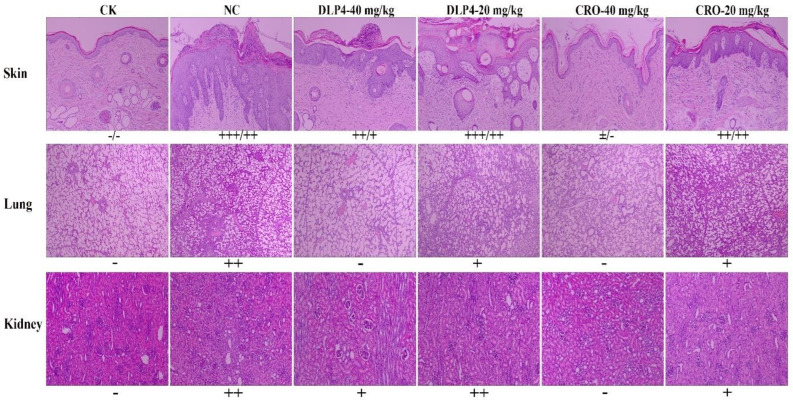
The protective effect of DLP4 on piglet skin, lung, and kidney induced by *S. hyicus* ACCC 61734. Histological assays of skin, lung, and kidney from piglet (magnifications, ×100) at 9 d. CK: the uninfected mice; NC: the infected piglet without treatment. The symbol “+” represents the degree of skin and organ damage; -: normal tissue; “+”: mild histological lesion; “++”: more severe histological lesions; “+++”: severe histological lesions; “±”: tissue lesions between mild and more severe; the skin includes the epidermis and dermis.

To further explore whether DLP4 can supply protection for piglets from bacterial infection, the degree of organ damage was determined. As shown in Figure 9, no pathological changes were observed in the skin, lung, and kidney in the control group. The histopathology of specimens obtained from piglets in the untreated group indicated that the epidermis and dermis of the skin were damaged after the invasion of *S. hyicus* ACCC 61734. The cuticle surface was covered by inflammatory exudate, and a large area of cuticle abscess appeared, accompanied by numerous necrotic inflammatory cells in the pus. The epidermal spinous layer thickened significantly, the granular layer disappeared, and the junctions of the epidermal cells were defective, showing diffuse “apoptosis”. In addition, the dermal papilla was a cluster-like extension; inflammatory cells infiltrated into the dermal connective tissue and deeper muscle layer, leading to fibrous tissue proliferation. Meanwhile, inflammatory cell infiltration and local fibrous hyperplasia were found in both lung and kidney tissues. In contrast, after treatment with DLP4, less damage was found in the skin, lung, and kidney, which were similar to those of CRO (40 mg/kg) group. All these data prove that DLP4 is effective in treating piglet EE caused by *S. hyicus* ACCC 61734 infection.

## 4. Discussion

Skin, as the first line of defense, can effectively protect various tissues and organs from physical, mechanical, chemical, and pathogenic microorganisms. Acantholysis is counted as one of the most dominating skin diseases, which mainly manifests as the failure of desmosomal adhesion with keratinocyte separation caused by genetic, autoimmune, or infectious causes [33]. Exfoliative superficial pyodermas (ESP), SSSS, and EE are all typical acantholysis caused by the hydrolyzation of protease released by *Staphylococcus* spp., and the therapeutic potential of these skin disorders has recently attracted much attention [34]. However, these diseases are difficult to treat. Represented by EE, there is even very barely objective evidence for the optimum treatment of EE, which is mainly due to the following aspects: firstly, the danger of EE lies in its destruction of the skin barrier, creating opportunities for the invasion of other pathogens, easily causing secondary infections, and even cause severe sepsis [9]. Secondly, the presence of biofilm in 78–90% of wounds, especially in *Staphylococcus species*, has the dual effect of prolonging inflammation and inhibiting re-epithelialization, thereby hampering wound healing [16,35,36,37,38]. Lastly, with the wide use of antibiotics in food-producing animals, more and more isolated bacteria developed resistance toward various antibiotics, which may be transmitted to humans, adversely affecting human health and limiting the choice of therapeutic drugs [39]. Therefore, the development of new and highly effective antimicrobial agents is an urgent priority for humans and animals. AMPs, as a new antibacterial agent, have attracted wide attention due to their superior antibacterial activity. In this study, we systematically and comprehensively studied the effect of insect defensin DLP4 on *S. hyicus* ACCC 61734 biofilm and its potential efficacy against EE in piglets.

Biofilm provides a favorable habitat for *S. hyicus*, which can effectively evade the effects of antibiotics and the immune system, leading to recurrent infection. Numerous studies have shown that AMPs such as IDR-1018, LL-37, SHAP1, Def-1, NZ2114, and P2 hold great promise for biofilm inhibition and wound healing in *Staphylococcus* spp. [21,40,41,42]. In this study, it was first demonstrated that *S. hyicus* is capable of forming biofilm, which poses difficulties in the treatment of EE. Further studies have shown that DLP4 can inhibit the formation of early biofilm and effectively eradicate mature biofilm. The mature biofilm clearance rate of DLP4 (128 µg/mL) towards *S. hyicus* NCTC 10350 and *S. hyicus* ACCC 61734 reached 90%, which was superior to that of CRO, indicating that DLP4 possesses better biofilm clearance ability (Figure 4). Meanwhile, studies have shown that bacteria can evade the immune system and antibacterial drugs by forming persisters in biofilm, thus causing persistent infections in the organism and hindering the effective treatment of bacterial infections [43,44]. Therefore, antimicrobial studies must focus not only on the ability to remove biofilm but also on verifying the antibacterial activity against the persistent bacteria in the biofilm. The present study showed that DLP4 (32 × MIC) inhibited 87.2–90.3% of persisters in the biofilm (Figure 5C,D), which was much better than that of CRO. This may be attributed to the cationic nature of DLP4, which gives it the ability to disrupt the biofilm, thereby facilitating the penetration of the drug into the biofilm and then enhancing the antimicrobial activity against persister in biofilm [45,46].

To evaluate the therapeutic effect of DLP4, a piglet EEmodel was established. As shown in Figure 6A, after infection with *S. hyicus* ACCC 61734 for 1 d, piglets showed obvious oily exudation and skin peeling. This is inconsistent with previous symptoms of *S. hyicus* ACCC 61734 infection in mice, which may be related to the pathogenic mechanism of *S. hyicus* ACCC 61734 [2]. A previous study showed that EE shared similar pathological features with SSSS, which may account for the generation of similar toxins from *S. hyicus* and *S. aureus* [33]. As shown in Figure 9, after infection with *S. hyicus* ACCC 61734, the histopathological findings of piglet skin showed typical skin pathological features of EE, the Dsg1 between the keratinocytes of the epidermal granular layer of the skin was digested, the connection between the cells was broken, and the skin tissue section showed obvious gaps (Figure 9), which mainly attributed to the following development and progression of EE. When *S. hyicus* ACCC 61734 invades piglets, protein A can inhibit the phagocytic ability of phagocytes, effectively reduce the piglets’ ability to resist bacteria [47,48] and make *S. hyicus* ACCC 61734 colonize the skin surface successfully and then the ExhD, an exotoxin secreted by *S. hyicus* ACCC 61734, plays the role of molecular scissors to specifically digest epidermal Dsg1, so that bacteria can invade from the skin surface to the deep layer and even to the blood circulation system (Figure 6F), causing sebum overflow, and forming dermatitis and viscera lesions (Figure 6). However, the exotoxin was species-specific in its digestion of Dsg1, which caused only abscesses in mice after infection [2]. Meanwhile, to evaluate the therapeutic effect of DLP4 as objectively as possible, we have initially established a clinical scoring system of EEaccording to the SCORAD, in which the extent criteria is determined based on the percentage of each part in the body surface, and the weight given to each item was fully referenced to SCORAD [31]. The results showed that EE was relieved to varying degrees after treatment, but at the same time, different individual score results did not show significant differences, which may illustrate the effectiveness of the scoring system. However, it also needs to be extensively validated and refined in the future.

Numerous studies indicated that AMP, as a promising antibacterial candidate, not only has high-efficiency bactericidal functions but also has immunoregulatory functions [49,50,51]. In this study, after infection with *S. hyicus* ACCC 61734, piglet quickly mobilized their own humoral and cellular immunity and regulated the release of cytokines to protect them from lethal injury. After treatment with DLP4, the levels of TNF-α, IL-10, and IL-6 decreased slightly (Figure 8A), while the levels of IL-12p40p70 and GM-CSF only increased slightly. TNF-α, as the initial factor of the cytokine cascade reaction, is commonly considered to be one of the dominant mediators of sepsis [52,53]. When threatened by *S. hyicus* ACCC 61734, macrophages, mast cells, and dendritic cells in piglets were activated, which can further promote the differentiation of T cells and the release of TNF-α, IL-6, and IL-12p40p70. Simultaneously, bone marrow hematopoietic stem cells released GM-CSF under the stimulation with *S. hyicus* ACCC 61734, which plays a vital role in nonspecific cellular immunity against infection. The ability of DLP4 to effectively protect piglets from EE may be due to its ability to balance pro- and anti-inflammatory factors.

In addition to acting as a physical barrier, keratinocytes in the epidermis express the Toll-like receptor TLR2 [54,55,56], which recognizes the pattern-associated molecular patterns (PAMPS: Lipoprotein, lipopeptide, peptidoglycan, lipoteichoic acid) of Gram-positive bacteria, and activate MyD88, IRAK serine/threonine kinase family and transcription factor NF-κB, thus initiating early skin innate immune response [57,58]. The regulation of DLP4 on the NF-κB pathway was further investigated by western blot. The results showed that DLP4 inhibited the expression of p-IκB and p-p65 and down-regulated the NF-κB pathway, which is consistent with the results of DLP4-derived peptide ID13 negatively regulated p-p65 and down-regulated the NF-κB pathway [26]. However, it remains unclear whether DLP4 exerts regulatory effects on innate immunity by modulating the release of AMPs. Consequently, further conclusions need to be verified, especially on the time spatial relationship between AMPs and immunomodulation, which is almost a common bottleneck and blind spot in the pharmacological field of AMPs; it should be a priority direction of investigation in subsequent AMPs studies [18,59,60].

## 5. Conclusions

**Figure 10:**
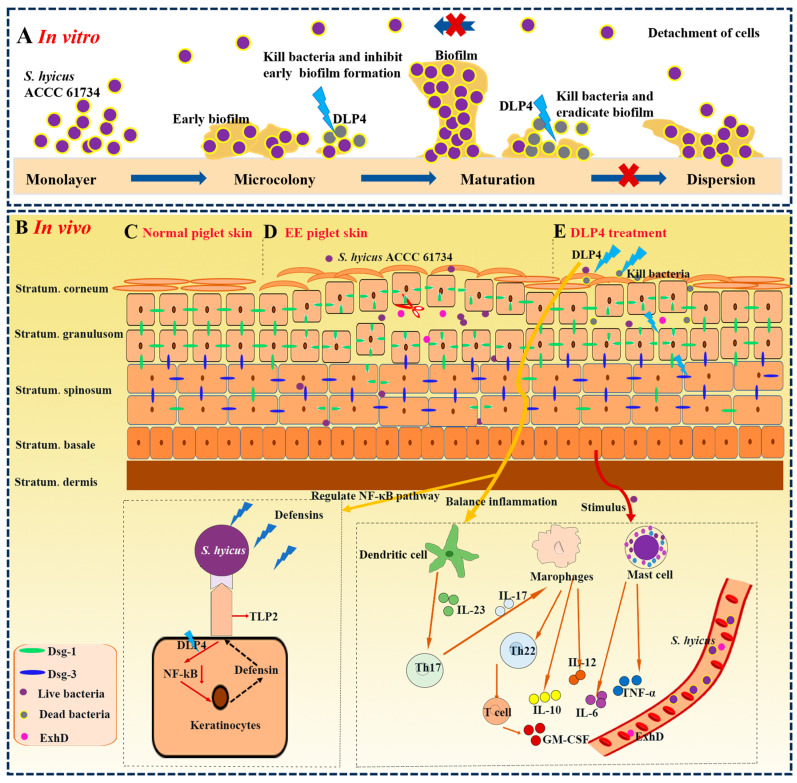
Inhibitory effect of DLP4 on the *S. hyicus* biofilm and potential efficacy on EE caused by *S. hyicus* ACCC 61734 in piglets. (**A**) Inhibitory effect of DLP4 on *S. hyicus* ACCC 61734 biofilm. DLP4 treats *S. hyicus* ACCC 61734 infections by bactericidal action, inhibition of early biofilm formation, and eradication of mature biofilm. (**B**) The potential efficacy of DLP4 on EE in piglets caused by *S. hyicus* ACCC 61734. (**C**) Normal piglet skin. (**D**) After infection of *S. hyicus* ACCC 61734, protein A on the bacterial cell wall inhibits the ability of phagocytic cells, thereby facilitating bacterial colonization and further causing EE. In EE, blisters influence only the surface of the skin, but not the mucosa or deeper layer, which can be perfectly interpreted by the specific elimination of exfoliation toxins on desmoglein and the different distribution of specific desmoglein in the skin epidermis. The ExhD released from *S. hyicus* ACCC 61734 can only selectively digest Dsg-1, which exists in all skin layers, whereas Dsg-3 remains impervious and only exists in the deeper skin layer. (**E**) Piglets skin of treatment group: the immune regulation of DLP4 after *S. hyicus* infection. Red arrow: promotion, black dashed arrow: potential inhibition, orange arrow: pathway.

In brief, we systematically investigated the in vitro and in vivo effects of DLP4 against *S. hyicus* ACCC 61734. The results showed that DLP4 exhibited potent antibacterial activity against *S. hyicus* ACCC 61734. DLP4 can effectively inhibit the formation of early biofilm of *S. hyicus* ACCC 61734, eradicate mature biofilm, and kill vancomycin-resistant persistent bacteria in the mature biofilm. In vivo studies showed that DLP4 effectively alleviated EE in piglets caused by *S. hyicus* ACCC 61734 by downregulating the NF-κB pathway, balancing cytokines, and inhibiting bacterial proliferation, which can be used as a potential therapeutic agent for the treatment of EE. Meanwhile, as porcine skin is similar to human skin in anatomy, physiology, and biochemistry, this study provides data supporting the treatment of human skin infections caused by *S. hyicus* (Figure 10). 

## Figures and Tables

**Table 1:** Criteria for the degree of exudative epidermitis symptoms.

Position	Proportion (%)
Head	20
Trunk	50
Fore limb	9
Hind limb	15
Genitalia	6

Note: 1% of area represents 1 point.

**Table 2:** The MIC assay of DLP4 and antibacterial agents against *S. hyicus* and *S. sciuri*.

Strains	DLP4		Ceftriaxone Sodium		Ceftiofur	
	μM	μg/mL	μM	μg/mL	μM	μg/mL
*S. hyicus* NCTC 10350	0.92	4	3.02	2	6.04	4
*S. hyicus* ACCC 61734	0.92	4	6.04	4	12.08	8
*S. sciuri* FRI 18	0.46	2	12.08	8	12.08	8
*S. sciuri* FRI 5	0.46	2	1.51	1	1.51	1

**Table 3:** The corresponding parameters of antibacterial activity of DLP4 and CRO against *S. hyicus* in vitro.

Strains	Antibacterial Agents	Emax (Log_10_ CFU/mL, 95% CI)	EC_50_ (μM)	R^2^
*S. hyicus* NCTC 10350	DLP4	−4.966 (−5.151 to −4.780)	5.580	0.987
	CRO	−4.752 (−5.004 to −4.459)	8.413	0.985
*S. hyicus* ACCC 61734	DLP4	−4.855 (−4.972 to −4.739)	3.158	0.994
	CRO	−4.891 (−4.980 to −4.803)	4.406	0.997

Note: Emax: maximum relative effectiveness, used to speculate when infinite high drug concentration, 24 h after the strains measured relative to the initial strains of the lower order of magnitude; CI: confidence interval; EC_50_: Drug concentration calculated by the Hilton equation when the bacterial volume is decreased by half. R^2^ represents the correlation coefficient.

**Table 4:** The clinical EE score of infected piglets.

Clinical EE Score (Mean)	Group					
	CK	NC	DLP4-40 mg/kg	DLP4-20 mg/kg	CRO-40 mg/kg	CRO-20 mg/kg
1 d	0	6.467	4.885	4.996	5.948	6.871
Within-observer SD	0	1.234	1.334	1.193	1.679	1.373
Between-observer SD	0	0.300	0.185	0.284	0.288	0.451
4 d	0	29.383	19.682	27.608	19.758	26.414
Within-observer SD	0	2.340	1.110	1.123	2.589	2.096
Between-observer SD	0	1.283	0.938	0.652	0.982	1.186
9 d	0	48.467	21.716	36.233	21.231	23.897
Within-observer SD	0	2.808	1.499	2.501	2.424	2.683
Between-observer SD	0	0.833	0.230	0.533	0.089	0.017

## Data Availability

The original contributions presented in the study are included in the article; further inquiries can be directed to the corresponding author(s).
